# Neuroprotective and anti-oxidant effects of caffeic acid isolated from *Erigeron annuus *leaf

**DOI:** 10.1186/1749-8546-6-25

**Published:** 2011-06-24

**Authors:** Chang-Ho Jeong, Hee Rok Jeong, Gwi Nam Choi, Dae-Ok Kim, Uk Lee, Ho Jin Heo

**Affiliations:** 1 Department of Food Science and Biotechnology, Institute of Life Sciences and Resources, Kyung Hee University, Yongin 446-701, Korea; 2 Department of Food Science and Technology, Institute of Agriculture and Life Science, Gyeongsang National University, Jinju 660-701, Korea; 3 Department Special Purpose Trees, Korea Forest Research Institute, Suwon 441-847, Korea

## Abstract

**Background:**

Since oxidative stress has been implicated in a neurodegenerative disease such as Alzheimer's disease (AD), natural antioxidants are promising candidates of chemopreventive agents. This study examines antioxidant and neuronal cell protective effects of various fractions of the methanolic extract of *Erigeron annuus *leaf and identifies active compounds of the extract.

**Methods:**

Antioxidant activities of the fractions from *Erigeron annuus *leaf were examined with [2,2-azino-bis(3-ethylbenz thiazoline-6-sulfonic acid diammonium salt)] (ABTS) and ferric reducing antioxidant power (FRAP) assays. Neuroprotective effect of caffeic acid under oxidative stress induced by H_2_O_2 _was investigated with [3-(4,5-dimethythiazol-2-yl)-2,5-diphenyl tetrazolium bromide] (MTT) and lactate dehydrogenase (LDH) assays.

**Results:**

This study demonstrated that butanol fraction had the highest antioxidant activity among all solvent fractions from methanolic extract *E. annuus *leaf. Butanol fraction had the highest total phenolic contents (396.49 mg of GAE/g). Caffeic acid, an isolated active compound from butanol fraction, showed dose-dependent *in vitro *antioxidant activity. Moreover, neuronal cell protection against oxidative stress induced cytotoxicity was also demonstrated.

**Conclusion:**

*Erigeron annuus *leaf extracts containing caffeic acid as an active compound have antioxidative and neuroprotective effects on neuronal cells.

## Background

Oxidative stress refers to the imbalance between the production and removal of reactive oxygen species (ROS). Due to the reaction between ROS and macromolecules, generation of ROS can lead to damage or death of cells in various tissues [1]. Brain tissue is most vulnerable to oxidative stress due to its high glucose metabolism rate and low antioxidant defense enzyme level [2]. Natural antioxidants are promising candidates of chemopreventive agents for treating neurodegenerative diseases such as Alzheimer's disease (AD), cerebral ischemia and Parkinson's disease (PD) [3].

About 18 million people in the world suffer from AD, the number of which is expected to reach 34 million by 2025 [4,5]. Characterized by loss of memory and cognition, AD is one of the most serious health threats in aging societies. In AD patients, who have high sensitivity to ROS, accumulated intracellular hydrogen peroxide (H_2_O_2_) induces membrane lipid peroxidation, and sometimes even caspases [4]. Brains of patients suffering from AD are subjected to an increase of free radical damage due to oxidative stress [6]. Many phenolics protect neuronal cells from oxidative stress induced by ROS or amyloid-β protein which may be related to the pathogenesis of AD [7]. Some phytochemicals from natural plant sources such as fruits and vegetable may reduce the risk of AD because of their antioxidant properties [8]. Epidemiological observation shows that the increase of antioxidant uptake is inversely correlated to the risk of AD incidence [9].

We focus on various fractions of the methanolic extract of *Erigeron annuus *(*Yinianpeng*) leaf for antioxidant and neuronal cell protective potentials. *E. annuus*, which belongs to the Compositae family, is widely distributed in urban and rural areas of Korea and China. *E. annuus *has been used in Chinese medicine for treating indigestion, enteritis, epidemic hepatitis and hematuria [10]. Phytochemicals from this plant have been isolated and reported such as γ-pyranone derivatives, flavonoids, triterpenoids [11], phenolic derivatives [12,13], cyclopentenone derivatives [14] and sesquiterpenenes [15]. *E. annuus *possesses antioxidant [16] antiglycation and rat lens aldose reductase inhibition activities [17]. Moreover, *E. annuus *is cytoprotective [18] and antidiabetic [19]. However, little is known about *E. annuus*' neuronal cell protective effects against oxidative stress.

This study examines antioxidant and neuroprotective effects of all fractions of the methanolic extract of *Erigeron annuus *leaf and identifies active compounds of the extract.

## Methods

### Chemicals

RPMI 1640 medium, fetal bovine serum (FBS), horse serum (HS) were purchased from Gibco BRL (USA). Unless specified otherwise, all materials used in this study were purchased from Sigma Chemical (USA), including 2,2-azino-bis(3-ethylbenz thiazoline-6-sulfonic acid diammonium salt) [(NH_4_)_2_ABTS], potassium persulfate, 2,4,6-tripyridyl-S-triazine (TPTZ), vitamin C, thiobarbituric acid, ferrous sulfate (FeSO_4_), hydrogen peroxide (H_2_O_2_), dimethyl sulfoxide (DMSO), penicillin, streptomycin, 2',7'-dichlorofluorescein diacetate (DCF-DA), 3-[4,5-dimethythiazol-2-yl]-2,5-diphenyl tetrazolium bromide (MTT) assay kit and lactate dehydrogenase (LDH) assay kit.

### Plant extraction

*Erigeron annuus *leaves were collected from Jinju, Korea in September 2009 and were authenticated by the Institute of Agriculture and Life Sciences, Gyeongsang National University where voucher specimens were deposited. Samples were washed with running tap water before chopped into pieces. They were then oven-dried at 40°C for two days and ground to powder which was stored at -20°C until use. Organic solvent fractions of the methanolic extract of *E. annuus *were obtained as follows. Powder of *E. annuus *(50 g) was suspended and extracted with 500 ml of methanol at 70°C for two hours. The extracts were filtered through Whatman No. 2 filter paper (Whatman International, UK) and evaporated to dryness. The crude extracts were then extracted successively with chloroform, butanol and water to yield the corresponding chloroform (37.13%), butanol (15.19%) and water (47.68%) fractions.

### Determination of total phenolics

Total phenolics were determined by spectrophotometric analysis [20]. Total phenolics in organic solvent fractions of *E. annuus *extracts were expressed as milligrams of gallic acid equivalents (mg GAE/g) of sample.

### ABTS radical scavenging activity

2,2-azino-bis(3-ethylbenz thiazoline-6-sulfonic acid diammonium salt) [(NH_4_)_2_ABTS] was dissolved in water to make a concentration of 7 mM. ABTS^+ ^was produced through reacting the ABTS stock solution with 2.45 mM potassium persulfate (final concentration) and allowing the mixture to stand in the dark at room temperature for 12-16 hours before use. For the study of samples, the ABTS stock solution with 2.45 mM potassium persulfate was diluted with phosphate-buffered saline 5 mM, pH7.4 to obtain an absorbance of 0.70 at 734 nm. After addition of 980 μl of diluted ABTS to 20 μl of sample, the absorbance reading was taken five minutes after the initial mixing [20]. Vitamin C was used as the positive control. This activity was measured as percent ABTS scavenging calculated as % ABTS scavenging activity = [1 - (A_sample _- A_control_)/A_control_] × 100

### FRAP

The ferric reducing antioxidant power (FRAP) assay was developed by Jeong *et al. *[20]. Briefly, 1.5 ml of working, pre-warmed 37°C FRAP reagent (10 volumes 300 mM/L acetate buffer, pH3.6 + one volume of 10 mM/L 2,4,6-tripyridyl-S-triazine in 40 mM/L HCl + one volume of 20 mM/L FeCl_3_) was mixed with 50 μl of the test sample and standards. The mixture was vortexed and read against a reagent (blank at a predetermined time after sample-reagent mixing) at 593 nm absorbance. The test was performed at 37°C and the window of 0-4 minute reaction time was used. Vitamin C was used as the positive control. Reduction of the ferric-tripyridyltriazine to the ferrous complex formed an intense blue color which was measured at a wavelength of 593 nm. Intensity of the color is related to the amount of antioxidant reductants in the samples.

### Identification and quantification of active compounds

The most active fraction was determined with various assays. After assays, the butanol fraction was divided into 32 sub-fractions (BF1-BF32) by column chromatography with silica-gels (230-400 mesh, Merck, Germany) eluted with chloroform/methanol (gradient elution: 99/1 to 1/1). Compound 1 as an active compound was isolated and purified from sub-fraction BF17 with high performance liquid chromatograph (HPLC) on an Agilent instrument (1100 series, USA) with a 250 mm × 4.6 mm, 5 μm C_18 _column (Shiseido, Japan). Mobile phase consisted of acetonitrile: acetic acid: methanol: water (113:5:20:862, v/v/v/v). Flow rate was 1.0 ml per minute with an injection volume of 20 μl. Compounds were detected through monitoring the elution at 280 nm. Compound 1 was purified by preparative TLC with chloroform/methanol (4:1, v/v). NMR data including ^1^H and ^13^C spectra of Compound 1 dissolved in CD_3_OD were determined with a 500 MHz spectrometer (Bruker, Germany).

### Inhibition of lipid peroxidation assay with mouse brain homogenates

This assay was carried out according to the method described by Chang *et al. *[21]. The brain of young adult male *Balb/c *mice were dissected and homogenized in ice-cold Tris-HCl buffer (20 mM, pH7.4) to produce a 1/10 homogenate. The homogenate was centrifuged (Combi-514R, Hanil Co. Ltd., Korea) at 12,000 × *g *for 15 minutes at 4°C. Aliquots (0.1 ml) of the supernatant were incubated with the test samples in the presence of 10 μM FeSO_4 _(0.1 ml) and 0.1 mM vitamin C (0.1 ml) at 37°C for one hour. The reaction was terminated by the addition of 0.1 ml trichloroacetic acid (TCA) (28%, w/v) and 0.3 ml thiobarbituric acid (TBA) (1%, w/v) in succession; the solution was then heated at 100°C. After 15 minutes, the color of the MDA-TBA complex was measured at 532 nm. A well-known antioxidant, namely (+)-Catechin, was used as positive control. Three replicates were prepared for each test sample. The inhibition ratio (%) was calculated as follows.

### Neuronal cell culture

PC12 cells respond reversibly to nerve growth factor (NGF) by induction of the neuronal phenotype. PC12 cells (KCLB 21721, Korea Cell Line Bank, Korea) were propagated in Rosewell Park Memorial Institute (RPMI) 1640 medium containing 10% fetal bovine serum, 25 mM 4-(2-hydroxylethyl)-1-piperazineethanesulfonic acid (HEPES), 25 mM sodium bicarbonate, 50 units/ml penicillin and 100 μg/ml streptomycin.

### Measurement of intracellular oxidative stress

Levels of intracellular ROS were determined by 2',7'-dichlorofluorescein diacetate (DCF-DA) assay [22]. Briefly, cells (10^4 ^cells/well on 96-well) were treated for 10 minutes with the indicated concentrations of the caffeic acid isolated from butanol fraction of *E. annuus *or vitamin C. The cells were then treated with or without 200 μM H_2_O_2_for two hours. At the end of the treatment, cells were incubated in the presence of 50 μM DCF-DA in phosphate buffered saline (PBS). Fluorescence was then quantified on a TECAN fluorometer (SER-NR 94572, USA) with 485 nm excitation and 530 nm emission filters.

### Protective effect on oxidative stress

MTT reduction assay was determined with an *in vitro *toxicology assay kit (TOX-1, Sigma Co, USA). Neuronal PC12 cells were plated at a density of 10^6 ^cells/well on 96-well plates in 100 μl of RPMI. The cells were pre-incubated with caffeic acid isolated from butanol fraction of *E. annuus *for 48 hours before H_2_O_2 _(200 μM) was added. The cells were treated with or without H_2_O_2 _for two hours. The amount of MTT formazan product was determined through measuring absorbance with a microplate reader (680, Bio-Rad, Japan) at a test wavelength of 570 nm and a reference wavelength of 690 nm.

Neuronal PC12 cells were precipitated through centrifugation (Combi-514R, Hanil Co. Ltd., Seoul, Korea) at 250 × *g *for four minutes at room temperature, 100 μl of the supernatants was transferred into new wells. LDH was determined with an *in vitro *toxicology assay kit (TOX-7, Sigma Co, USA). Damage of the plasma membrane was evaluated through measuring the amount of the intra-cellular enzyme LDH released into the medium.

### Statistical analysis

All data were expressed as mean ± SD (*n *= 3). Data were analyzed with one-way of variance (ANOVA) and Duncan's multiple range test in SAS (8.2 version, SAS Institute, USA).

## Results and discussion

### Total phenolics and antioxidant activities of various fractions of the methanolic extract of E. annuus

Expressed as gallic acid equivalent (GAE), the total phenolics in various solvent fractions of the methanolic extract of *E. annuus *were determined according to the Folin-Ciocalteu method [20]. Total phenolic contents in butanol fraction were the highest (396.49 mg of GAE/g), followed by water fraction (241.87 mg of GAE/g) and chloroform fraction (107.34 mg of GAE/g) (Table 1). Many studies suggested that antioxidant activity of plants was likely related to redox properties of their phenolics behavior (*eg *as reducing agents, hydrogen donors and singlet oxygen quenchers) [23].

**Table 1:** Total phenolic contents and EC_50 _values (ABTS free radical scavenging assay) of their derived fractions of the methanolic extract of *E. annuus *leaf

Solvent fractions	EC_50 _(μg/ml)	Total phenolics (mg of GAE/g)
Chloroform	528.81	107.34 ± 1.87*
Butanol	250.00	396.49 ± 2.18
Water	304.76	241.87 ± 4.06**
Vitamin C	47.97	-

EC_50_: 50% effective concentration.

Results are presented as mean ± SD of three independent experiments; the letters (a-d) indicate statistically significant differences (*P = 0.025, ** *P *= 0.047).

**Figure 1:**
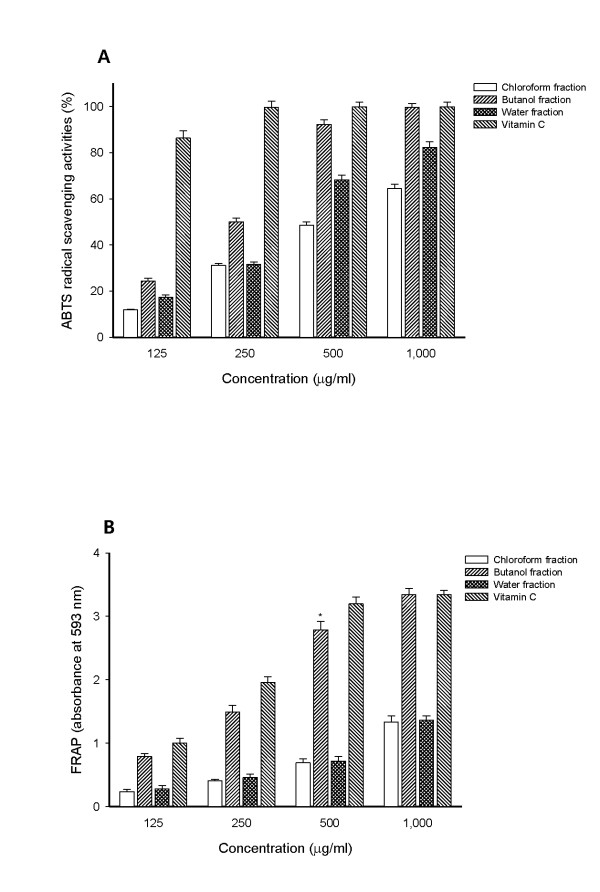
**(A) ABTS radical scavenging activities and (B) FRAP of fractions from the methanolic extract of *E. annuus *leaf**. Results are presented as mean ± SD of three independent experiments. (B) *P = 0.022, vs. positive control.

The ABTS radical scavenging activities of the various fractions of the methanolic extract of *E. annuus *were estimated through comparing the percentage inhibition of the formation of ABTS radicals by the various fractions and that of vitamin C. As shown in Figure 1A, the highest activity was observed in the butanol fraction and the water fraction also showed good inhibitory effects. In the presence of the 1,000 μg/ml test sample, the ABTS radical inhibition of organic solvent fractions decreased in the following order: butanol fraction (99.69%) > water fraction (82.32%) > chloroform fraction (64.48%). Vitamin C (positive control), a well-known natural antioxidant, showed 99.86% inhibition on the ABTS radical at a concentration of 500 μg/ml (Figure 1A).

The EC_50 _value of vitamin C, chloroform, butanol and water fractions were 47.97, 528.81, 250.00 and 304.76 μg/ml respectively (Table 1). Kim and Kim [16] found that 50% ethanol extract of whole *E. annuus *possessed significant ABTS radical scavenging activity with an EC_50 _value of 125 μg/ml.

Another antioxidant activity was studied through ferric reducing antioxidant power assay. Samples were used in a redox-linked reaction where the antioxidants in the sample acted as oxidants.. As shown in Figure 1B, the ferric reducing antioxidant power of various fractions of methanolic extract of *E. annuus *at 1,000 μg/ml were as follows: butanol fraction (absorbance value = 3.34) > water fraction (absorbance value = 1.36) > chloroform fraction (absorbance value = 1.34). Ferric reducing antioxidant power of the butanol fraction was the highest among all fractions and increased linearly with increasing concentrations. These results agreed to another study with similar correlations between total polyphenols and antioxidant activity [24].

### Identification and quantification of caffeic acid as an active compound

**Figure 2:**
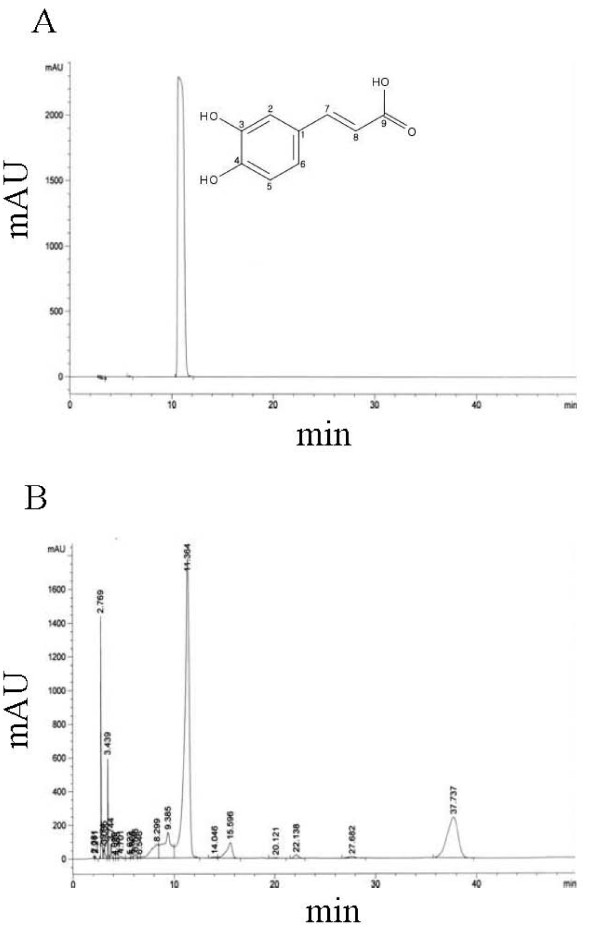
**(A) HPLC chromatogram of commercial standard and (B) caffeic acid isolated from the butanol fraction of *E. annuus *leaf**.

**Figure 3:**
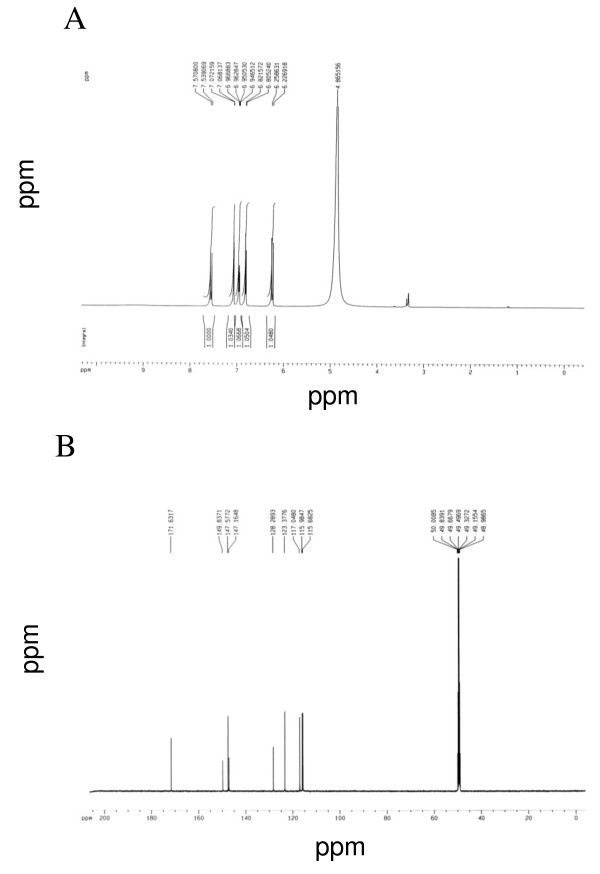
**(A) ^1^H-NMR and (B) ^13^C-NMR spectrum of caffeic acid isolated from the butanol fraction of *E. annuus *leaf**.

Among the column fraction of butanol fraction, BF17 had an excellent ABTS radical scavenging activity with an EC_50 _value of 112.26 μg/ml. To find out its active component, we isolated and identified Compound 1 as an active compound from BF17 using HPLC (retention time = 11.36 minutes) (Figure 2) and NMR spectrometry. Compound 1 was characterized as a caffeic acid with following characteristics: yellow amorphous solid: ESIMS *m/z *180; ^1^H NMR (CD_3_OD, 500 MHz) δ: 7.55 (1 H, d, J = 15.9 Hz, H-7), 7.07 (1 H, d, J = 2.0 Hz, H-2), 6.95 (1 H, dd, J = 8.2, 2.0 Hz, H-6), 6.81 (1 H, d, J = 8.2, H-5), 6.24 (1 H, d, J = 15.9 Hz, H-8); ^13^C-NMR (CD_3_OD, 125 MHz) δ 171.6 (C-9), 149.8 (C-4), 147.6 (C-7), 147.2 (C-3), 128.3 (C-1), 123.4 (C-6), 117.0 (C-5), 116.0 (C-8), 115.7 (C-2) (Figure 3). Spectral data of the isolated caffeic acid were in good agreement with the published values of standards [25]. HPLC quantification revealed that 3.68 μg of caffeic acid was in 1 mg of butanol fraction.

### Inhibition of lipid peroxidation and intracellular accumulation of ROS by caffeic acid

**Figure 4:**
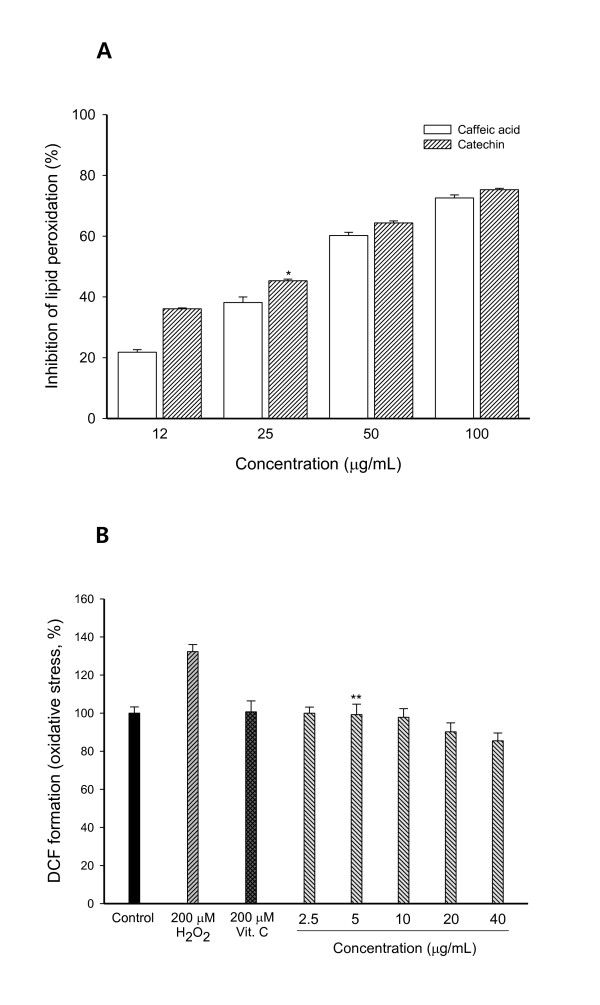
**Inhibition effect of caffeic acid isolated from butanol fraction of *E. annuus *leaf on both ferric ion and vitamin C-induced lipid peroxidation on mouse brain homogenates (A) and free radical production determined in the presence and absence of H_2_O_2 _in PC12 cell (B)**. Results are presented as mean ± SD of three independent experiments. (A) *P = 0.024, vs. positive control; (B) **P = 0.029, vs. positive control.

Inhibition of lipid peroxidation assay confirmed antioxidant activities of caffeic acid isolated from butanol fraction of *E. annuus *on both ferric ion and vitamin C-induced lipid peroxidation on mouse brain homogenates. Caffeic acid suppressed lipid peroxidation on mouse brain homogenates (Figure 4A). Caffeic acid showed less effectiveness than (+)-catechin at all concentrations; more than 50% of inhibitory activity of lipid peroxidation was observed at the concentration of 50 μg/ml. However, caffeic acid had an EC_50 _value of 38.43 μg/ml, compared to (+)-catechin (31.17 μg/ml). Previous studies indicated that caffeic acid had excellent antioxidant and neuroprotective effects [26]. These results suggested a potential use of the crude extract of *E. annuus *as well as the isolated compounds for treating neurodegenerative diseases such as AD.

To examine intracellular accumulation of ROS in PC12 cells used as neuronal cell model, we used 2',7'-dichlorofluorescein diacetate (DCFH-DA) probe which is freely permeable across cell membrane. DCFH-DA was hydrolyzed by cytosolic esterases to non-fluorescent dichlorofluorescein (DCFH). DCFH that interacted with ROS was oxidized to a highly fluorescent substance, namely 2',7'-dichlorofluorescein (DCF). Exposure of PC12 cells to H_2_O_2_for two hours resulted in a 132.28% increase of the ROS levels compared to control (Figure 4B). Pretreatment of PC12 cells by caffeic acid significantly prevented them from intracellular ROS accumulation in comparison to the PC12 cells treated only with H_2_O_2 _(control). Vitamin C is one of the naturally occurring major nutrients with antioxidant activity. PC12 cells had significantly lower oxidative stress than PC12 cells with treatments of H_2_O_2 _only (Figure 4B). This result suggested that caffeic acid isolated from butanol fraction of *E. annuus *with antioxidant activity might play an important role in reducing the oxidative stress.

### Protection of PC12 cells treated with by H_2_O_2 _caffeic acid

**Figure 5:**
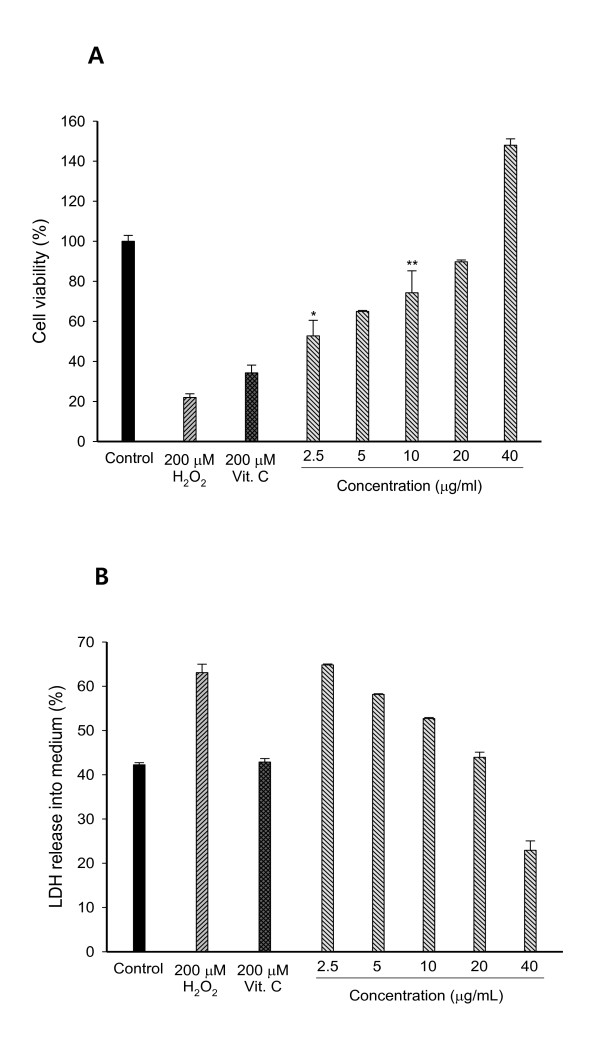
**(A) Protective effects of caffeic acid isolated from *E. annuus *leaf on hydrogen peroxide-induced neurotoxicity and (B) membrane damage in PC12 cell system**. PC12 cells were pretreated for 48 hours with various concentrations. After 48 hours, cells were treated with 200 μM H_2_O_2 _for two hours. Results are presented as mean ± SD of three independent experiments. (A) *P = 0.037, **P = 0.046, vs. positive control.

As shown in Figure 5A, the protection of PC12 cells increased dose-dependently with the concentrations at 2.5-40 μg/ml and reached the best protection *ie *148% of control group, at 40 μg/ml. Our results indicated that caffeic acid protected neuronal PC12 cells against H_2_O_2_-induced neurotoxicity.

As the neuronal plasma membrane is sensitive to oxidative stress, we measured the LDH activity released from apoptotic PC12 cells into the medium. A quantitative analysis of LDH activity can determine the percentage (%) of dead cells. Inhibition rates of caffeic acid isolated from *E. annuus *against H_2_O_2_-induced membrane damage at different concentrations were shown in Figure 5B. Treatment with 200 μM H_2_O_2 _caused an increase in LDH release into the medium (63.08%). Pretreatment with caffeic acid caused an inhibitory effect on LDH release with the highest inhibition (22.92%) at 40 μg/ml.

The phenolic hydroxyl groups of caffeic acid, particularly the ortho-hydroxy phenol group, were suggested to be a stable oxidation intermediate, the ortho-hydroxyphenoxyl radical that could quench free radicals [27]. These findings suggested that the strong antioxidant activities of caffeic acid decreased the H_2_O_2_-induced oxidative stress Oxidative damage is one of the neurotoxic mechanisms induced by H_2_O_2_. Early depletion of antioxidant compounds such as glutathione was considered important to the development of AD pathology [28]. Therefore, antioxidant activities of caffeic acid may provide neuroprotection against H_2_O_2_-induced toxicity. Future investigation is warranted to elucidate the cellular mechanism for the neuroprotection of *E. annuus *leaf phenolics, caffeic acid in particular.

## Conclusion

The butanol fraction had the highest antioxidant activity as revealed in the ABTS and FRAP assays. Moreover, caffeic acid decreased oxidative stress induced by H_2_O_2 _and demonstrated very strong antioxidant activities and neuronal cell protective effects. *E. annuus *leaf may be used as an anti-oxidant and chemopreventive agent to treat neurodegenerative disorders such as AD.

## Abbreviations

ABTS: 2,2-azino-bis(3-ethylbenz thiazoline-6-sulfonic acid); FRAP: ferric reducing antioxidant power; MTT: 3-[4,5-dimethythiazol-2-yl]-2,5-diphenyl tetrazolium bromide; LDH: lactate dehydrogenase; ROS: reactive oxygen species; AD: Alzheimer's disease; PD: Parkinson's disease; H_2_O_2_: hydrogen peroxide; TCA: trichloroacetic acid; TBA: thiobarbituric acid; MDA: malondialdehyde; NGF: nerve growth factor; DCF-DA: 2',7'-dichlorofluorescein diacetate; PBS: phosphate buffered saline

## Competing interests

The authors declare that they have no competing interests.

## Authors' contributions

CHJ and HJH designed the study. CHJ, GNC and HRJ conducted the experiments, analyzed the data and drafted the manuscript. DOK revised the manuscript. UL helped conduct the experiments. All authors read and approved the final version of the manuscript.
